# Specific gastrointestinal microbiota profiles in Chinese Tan sheep are associated with lauric acid content in muscle

**DOI:** 10.1186/s12866-023-03079-2

**Published:** 2023-11-08

**Authors:** Zhen LI, Ran Cui, Yu-Bei Wang, Ya-Biao Luo, Peng-Xiang Xue, Qi-Guo Tang, Mei-Ying Fang

**Affiliations:** Department of Animal Genetics and Breeding, National Engineering Laboratory for Animal Breeding, MOA Laboratory of Animal Genetics and Breeding, College of Animal Science and Technology, China Agricultural University, Beijing, 100193 China

**Keywords:** Tan sheep, Gut microbiota, Metagenomics, 16S rDNA

## Abstract

The biological mechanisms underlying meat quality remain unclear. Currently, many studies report that the gastrointestinal microbiota is essential for animal growth and performance. However, it is uncertain which bacterial species are specifically associated with the meat quality traits. In this study, 16S rDNA and metagenomic sequencing were performed to explore the composition and function of microbes in various gastrointestinal segments of Tan sheep and Dorper sheep, as well as the relationship between microbiota and meat quality (specifically, the fatty acid content of the muscle). In the ruminal, duodenal, and colonic microbiome, several bacteria were uniquely identified in respective breeds, including *Agrobacterium tumefaciens, Bacteroidales bacterium CF,* and several members of the family Oscillospiraceae. The annotation of GO, KEGG, and CAZYme revealed that these different bacterial species were linked to the metabolism of glucose, lipids, and amino acids. Additionally, our findings suggested that 16 microbial species may be essential to the content of fatty acids in the muscle, especially C12:0 (lauric acid). 4 bacterial species, including *Achromobacter xylosoxidans*, *Mageeibacillus indolicus*, and *Mycobacterium dioxanotrophicus*, were positively correlated with C12:0, while 13 bacteria, including *Methanobrevibacter millerae*, *Bacteroidales bacterium CF*, and *Bacteroides coprosuis* were negatively correlated with C12:0. In a word, this study provides a basic data for better understanding the interaction between ruminant gastrointestinal microorganisms and the meat quality traits of hosts.

**Supplementary Information:**

The online version contains supplementary material available at 10.1186/s12866-023-03079-2.

## Introduction

The gastrointestinal tract is a system with great regional diversity. Each segment of the intestine has a specialized function that regulates complex and diverse digestive, immune, metabolic, and endocrine processes [1]. Gastrointestinal microflora can influence fatty acid contents of the muscle through metabolites like bile acids and short-chain fatty acids. Indeed, a study conducted on Rongchang pigs (obese) and Yorkshire pigs (lean) revealed notable differences in their intestinal flora. To further investigate the impact of these microbial communities on metabolism, fecal bacteria transplantation was performed by transferring the intestinal flora from the two pig groups to germ-free mice, and the recipient mice displayed similar metabolic phenotypes to the respective donor pigs [2]. This study indicated the interaction between intestinal flora and the host. Another study found *Lactobacillus*, *Bifidobacterium*, and *Faecalibacterium* in the gut catabolize inulin to produce SCFAs, which act as signaling molecules to activate specific cell surface receptors of GPR43 and GPR41 to regulate lipid metabolism [3, 4]. In addition, Ghost and Shen et al. have shown that gut microbes regulate lipid metabolism by affecting the formation of bile acids as well as the distribution of secondary bile acids [5, 6]. Altogether, these studies demonstrate how intestinal bacteria play a crucial role in host metabolism.

With the improvement in living standards, mutton quality and flavor have gradually become the focus of consumer concern [7]. The formation of good meat quality traits is related to a variety of factors. It was found that Tan sheep muscles contained considerably more intramuscular fat (IMF) and essential fatty acids (EFA) than other sheep breeds [8, 9]. This may be one reason for the unique flavor of Tan sheep meat. Meanwhile, oleic acid enhances mitochondrial fatty acid β oxidation and promotes mitochondrial biosynthesis through the PPARα pathway, thus improving meat flavor [10]. Shao [11] found that the content of palmitoleic acid (C16:1) in Mongolian sheep was positively correlated with meat flavor. Through the activation of TLR4 signaling, lactic acid encourages the production of glycolytic muscle fibers, and the properties of these fibers determine the quality of the meat [12]. Many studies have found that the glycerol monolaurate (GML) may influence the nutritional characteristics of meat [13], and improve meat quality [14]. It indicates that fatty acids are an important factor affecting meat flavor.

However, there is little research on the link between gut microbiota and the fatty acid content of the sheep muscle. Therefore, taking Dorper sheep as the control group, the 16S rDNA and genome sequencing of different gastrointestinal segments of Tan sheep were carried out, which revealed the microbial community composition and function of different gastrointestinal segments of Tan sheep, and further found microbes that correlate with fatty acid content of the muscle. These data provide a foundation for analyzing the molecular mechanism of good meat quality traits of Tan sheep.

## Materials and methods

### Research site and sampling

This study included Tan sheep and Dorper sheep, which were bred in Ningxia, China. Each group consisted of 8 individuals. The management and feeding practices were the same for experimental sheep. Corn and soybeans were freely consumed, and Roughage, including alfalfa and corn stover, were fed ad libitum. The sheep received no medication or antibiotics during the experiment period, either through food or injections. At the age of 8 months, 6 sheep from each group were randomly chosen, and the luminal contents of the rumen, duodenum, jejunum, cecum, and colon were collected for 16S rDNA sequencing. 22 were chosen for metagenomic sequencing, including 4 samples from the rumen and colon, and 3 from the duodenum. In a previous publication, the fatty acid content of the longissimus muscle of the two sheep breeds was reported [8].

### DNA extraction, PCR amplification, and 16S rRNA gene sequencing

The microbial DNA was obtained using the PowerSoil DNA Isolation Kit (MoBio Laboratories, Carlsbad, CA). The genomic DNA's quality and purity were evaluated using 1% agarose gels and a NanoDrop2000 spectrophotometer. The V3-V4 hypervariable portion of the bacterial 16S rRNA gene was synthesized using the specific primer sets 338F and 806R, respectively (5’-ACTCCTACGGGAGGCAGCAG-3’) and 806R (5’-GGACTACNNGGGTATCTAAT-3’) [15]. Subsequently, the Miseq PE300 platform was used to sequence the amplicons that were produced.

### Bioinformatic analysis

A total of 60 samples from the rumen, duodenum, jejunum, colon, and cecum were used for 16S sequencing. Initially, we performed raw data filtering, removing sequences with a low-quality score (≤ 20) and those less than 120 bp. The remaining qualified tags were denoised into OTU (Operational Taxonomic Units) employing the unoise3 algorithm [16]. Additionally, all sequences were categorized into various taxonomic classifications using the BLAST, and the Silva138 database was utilized as a reference [17]. QIIME (v1.8.0) software was used to determine the indexes of diversity according to OTU data [18]. Furthermore, the relative abundance of the flora was visualized using R software (v3.6.0). To evaluate the overall difference among gastrointestinal segments of the two breeds, Principal Component Analysis (PCA) was conducted using the ade4 package [19], considering the OTU data derived from all samples [20].

### Metagenomic sequencing, assembly, and construction of the gene catalog

From the luminal contents of the rumen, duodenum, and colon, we obtained a total of 22 DNA samples. The DNA samples were randomly fragmented into small genomic fragments of approximately 300bp utilizing a Covaris ultrasonic analyzer. Terminal repair, a-tail addition, sequencing connector addition, purification, amplification by PCR, and other processes were all a part of the library preparation process. After the library met the requirements, Illumina HiSeq sequencing was carried out.

To ensure data quality, we utilized Fastp software [21] for raw data quality control. Bowtie2 software was used to remove host contamination during the comparison process [22]. The assembled hits were filtered using MEGAHIT to eliminate fragments smaller than 500 bp and obtain contig [23]. With the Prodigal, the contig sequences were exposed to Open Reading Frame (ORF) prediction [24]. Non-redundant sequences based on 95% consistency were obtained using CD-HIT software [25]. Kraken2 was utilized for species classification, with MiniKraken2 v1 8GB database [26]. A correction was performed using Braken [27], resulting in a species abundance table. Microbiota comparison and annotation were carried out in public databases such as KEGG and GO using HUMAnN3 [28]. CAZymes were annotated using dbCAN [29].

### Statistical analysis

All the data visualization was done using R software (v4.0.4). The ALPHA diversity of each gastrointestinal segment was calculated with the assistance of the Vegan and Picante packages. The Lefse approach was used to figure out the bacterial species and their functional capabilities in the gut microbiome between two sheep breeds. Applying the standards of LDA score > 2.0, allowed the discovery of differences in abundance between the two breeds [30]. Spearman's correlations were used to analyze the relationships between bacterial species and the functional capabilities of the gut microbiome as well as between bacterial species and the content of fatty acids in the longissimus muscle.

## Results

### The microbial sequencing and the diversity of bacteria

A total of 5,395,551 clean data were generated from the 16S rDNA sequencing analysis. These sequences contained 89,926 reads on average per sample (Table S1). It was discovered that 7,578 operational taxonomic units (OTUs) clustered with 97% sequence identity. We divided the OTUs into 539 genera, 274 families, 164 orders, 107 classes, and 52 phyla. After filtering poor-quality reads and host pollution, metagenomic sequencing of 22 samples produced an entire 319.7 Gb of clean data, with a mean of 14.53 Gb per sample.

**Fig. 1:**
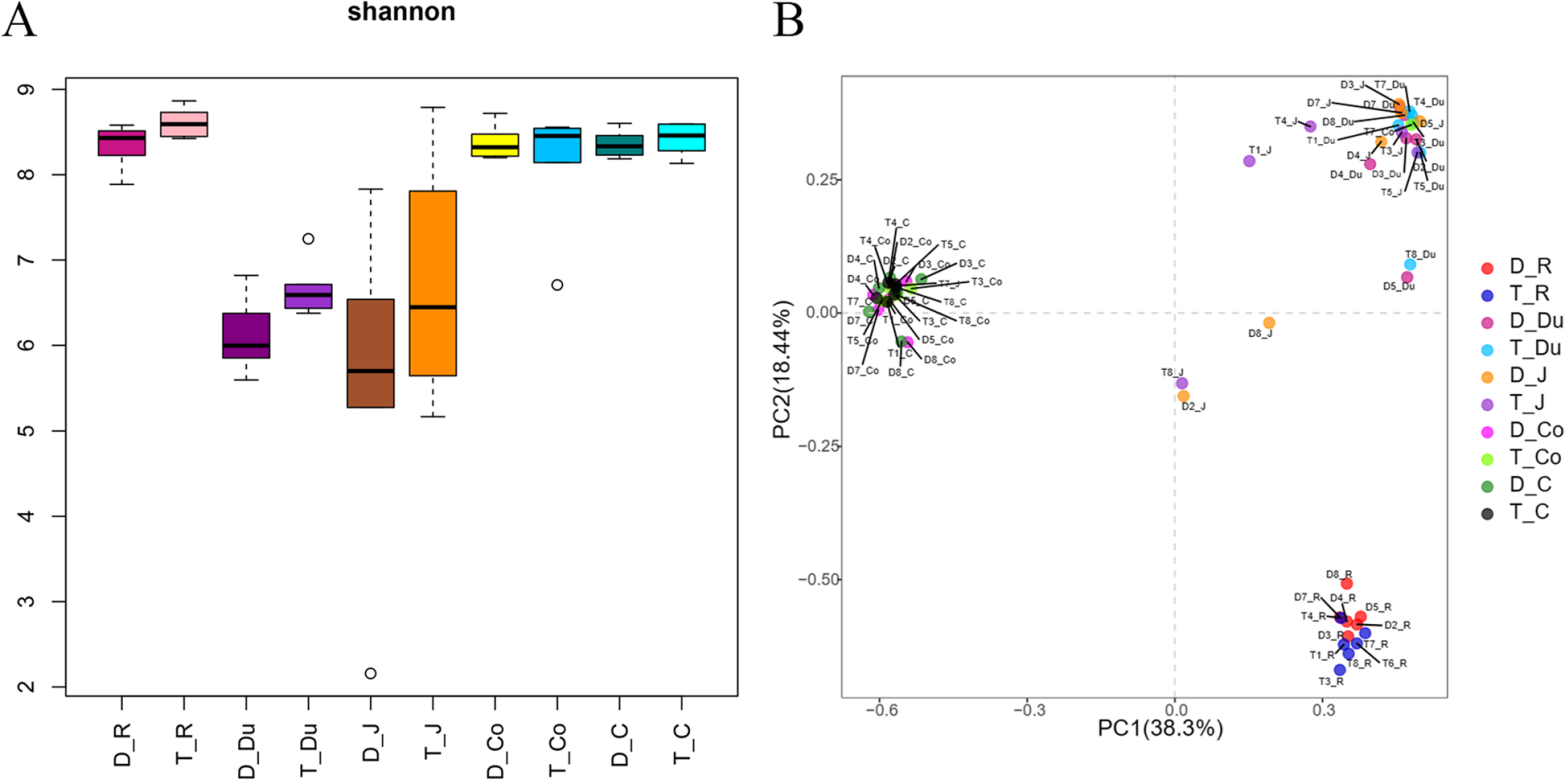
The diversity statistical analysis of the 16S sequencing. **A** Shannon index of OTU-level. **B** The principal component analysis (PCA) plot at the OTU level. D: Dorper sheep, T: Tan sheep, R: rumen, Du: duodenum, J: jejunum, Co: colon, C: cecum

At the OTU level, the Alpha diversity of different gastrointestinal segments in two sheep breeds was examined using 16S rDNA. Based on the Shannon Index, the Alpha diversity was found to be higher in the rumen and large intestine compared to the small intestine. Additionally, the Alpha diversity in Tan sheep showed a slight superiority over Dorper sheep (Fig. 1A, Table S2). Furthermore, the foregut, hindgut, and rumen have substantially distinct microbiota compositions, as shown by the principal coordinate analysis (PCA, Fig. 1B).

### Alterations in the microbial structure and function of the rumen, small and large intestines

**Fig. 2:**
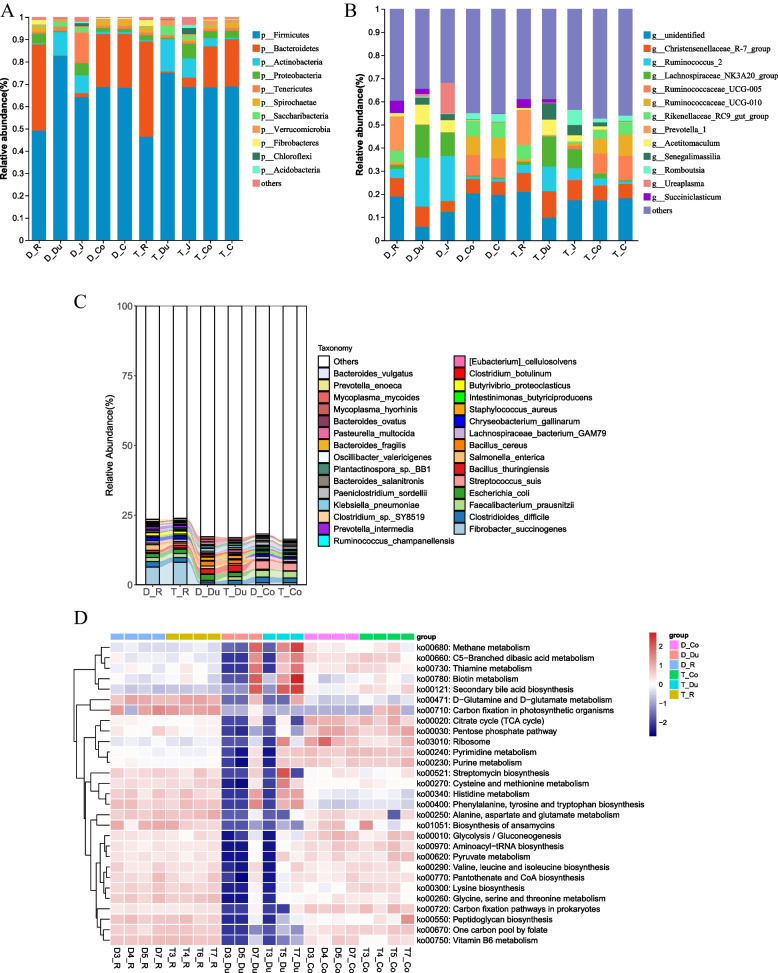
The result of the gut microbiome of the two breeds in terms of composition and functions. Phylum (**A**), genus (**B**), and species (**C**) levels of relative abundance of microbial communities in the rumen, foregut, and hindgut. **D** The KEGG activities of the microbiome of rumen, small and large intestine. The data of Phylum (**A**) and genus (**B**) levels of relative abundance of microbial communities are obtained from the 16S. Species (**C**) levels of relative abundance and the KEGG activities (**D**) are obtained from the metagenomic data. D: Dorper sheep, T: Tan sheep, R: rumen, Du: duodenum, J: jejunum, Co: colon, C: cecum

The relative abundance study of phyla, genera, and species revealed distinct microbial structures in the rumen, small intestine, and large intestine of the sheep. In this study, the small intestine represents the duodenum and jejunum, and the large intestine represents the colon and cecum. However, Firmicutes were the absolute dominant phyla in the gastrointestinal tract in both sheep breeds (Fig. 2A).

The rumen primarily contained *Prevotella1*, *Christensenellaceae R-7 group*, *Succiniclasticum*, *Rikenellaceae RC9 gut group*, and *Ruminococcus2* as the dominant bacteria at the genus level (Fig. 2B, Table S3). In the foregut, the dominant bacteria consisted of *Ruminococcus2*, *Lachnospiraceae NK3A20 group*, *Achristensenellaceae R-7 group*, and *Acetitomaculum* (Fig. 2B, Table S3). The hindgut exhibited dominant genera such as *Ruminococcaceae UCG-005*, *Ruminococcaceae UCG-010*, *Rikenellaceae RC9 gut group*, and *Christensenellaceae R-7 group* (Fig. 2B, Table S3).

According to the metagenomic analysis, the rumen was found to be dominated by species such as *Prevotella ruminicola*, *Fibrobacter succinogenes*, *Clostridioides difficile*, *Butyrivibrio fibrisolvens*, *Escherichia coli, and Methanobrevibacter millerae* (Fig. 2C). In the duodenum, the dominant species were *Methanobrevibacter millerae*, *Methanobrevibacter sp. YE315*, *Bacillus thuringiensis*, *Methanobrevibacter olleyae*,

*Escherichia coli*, and *Methanobrevibacter ruminantium*. In the colon, the dominant species included *Turicibacter_sp.H121*, *Streptococcus suis*, *Faecalibacterium prausnitzii*, *Clostridioides difficile*, *Oscillibacter sp. PEA192,* and *Clostridioides difficile*.

Furthermore, we determined distinct functional features in the rumen, foregut, and hindgut microbiomes. The rumen microbiome showed high enrichment of pathways such as valine, leucine, and isoleucine biosynthesis, alanine, aspartate, and glutamate metabolism, D-glutamine and D-glutamate metabolism, and aminoacyl-tRNA biosynthesis. The duodenum microbiome, on the other hand, exhibited enrichment in pathways like valine, leucine, and isoleucine biosynthesis, C5-branched dibasic acid metabolism, secondary bile acid biosynthesis, aminoacyl-tRNA biosynthesis, and methane metabolism. In the colon microbiome, pathways including valine, leucine, and isoleucine biosynthesis, aminoacyl-tRNA biosynthesis, C5-branched dibasic acid metabolism, alanine, aspartate, and glutamate metabolism, and TCA cycle were highly enriched (Fig. 2D).

### Differences in rumen microbial composition and function between Tan sheep and Dorper sheep

**Fig. 3:**
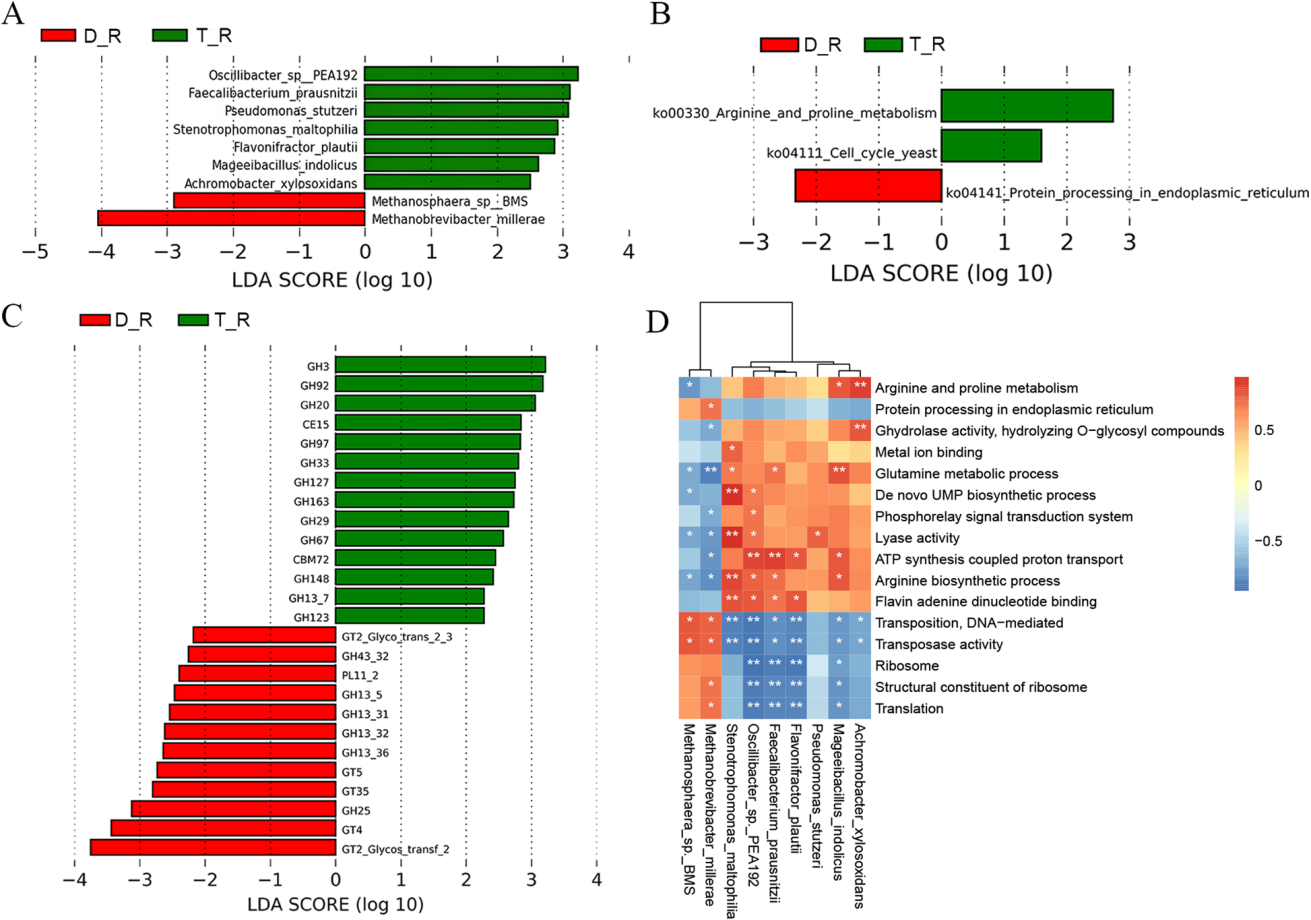
Differences in rumen microbial composition and function among two sheep breeds from metagenomic data. **A** LEfSe Analysis of ruminal microbiota among two sheep breeds at the species level. **B** The unique KEGG activities of different microflora of the two breeds. **C** Function terms of ruminal microbes by CAZy among two sheep breeds. **D** Heatmap of correlation between rumen microbiota and differential pathways. D: Dorper sheep, T: Tan sheep, R: rumen, Du: duodenum, Co: colon

Metagenomic sequencing was carried out on the rumen, duodenum, and colon of two sheep breeds to evaluate changes in microbial composition and function in various parts of the gastrointestinal tract. In the rumen contents, 9 bacterial species were observed with significant variations between Tan sheep and Dorper sheep. *Faecalibacterium prausnitzii*, *Oscillibacter sp. PEA192*, *Pseudomonas_stutzeri*, and *Stenotrophomonas maltophiliain* of Tan sheep were found to be significantly higher in Tan sheep, while *Methanobrevibacter millerae* and *Methanosphaera sp. BMS* were significantly lower compared to Dorper sheep (Fig. 3A).

According to KEGG functional analysis, the rumen in Tan sheep had enriched arginine and proline metabolic pathways (Fig. 3B). Furthermore, GO terms analysis (Fig. S1) identified 14 distinct pathways that differed between the two groups. In Tan sheep, it is primarily enriched in hydrolase activity, ATPase activity, glutamine metabolism, arginine synthesis, and phosphorus signal transduction system. Correlation analysis demonstrated differences between different bacteria species and pathways in the rumen such as *Achromobacter xylosoxidans* were positively correlated with arginine and proline metabolism (Fig. 3D). Most of the differentially abundant microbiota belonged to Oscillibacter, which showed positive correlations with ATP synthesis, arginine synthesis, and Flavin adenine dinucleotide binding. Conversely, *Methanobrevibacter millerae* exhibited a negative correlation with the glutamine metabolism process.

Moreover, we examined the CAZyme profiles of f Tan sheep and Dorper sheep in the rumen in the rumen. 26 CAZymes demonstrated quite distinct abundances among the two sheep breeds, with 14 of these CAZymes being enriched in Tan sheep and involved in xylan, glucan, mannose, and amylase. On the other hand, the ruminal microbiome of Dorper sheep exhibited higher abundances of 12 CAZymes primarily associated with the metabolism of arabinose, glycogen, sucrose, and bacterial capsule biosynthesis (Fig. 3C). Correlation analysis between the species and CAZymes indicated the contribution of the different bacteria to the changes in CAZymes (Fig. S2). The different bacteria led to the alterations in CAZymes, according to an association study between the species and CAZymes (Fig. S2).

### Differences in duodenal microbial composition and function between Tan sheep and Dorper sheep

**Fig. 4:**
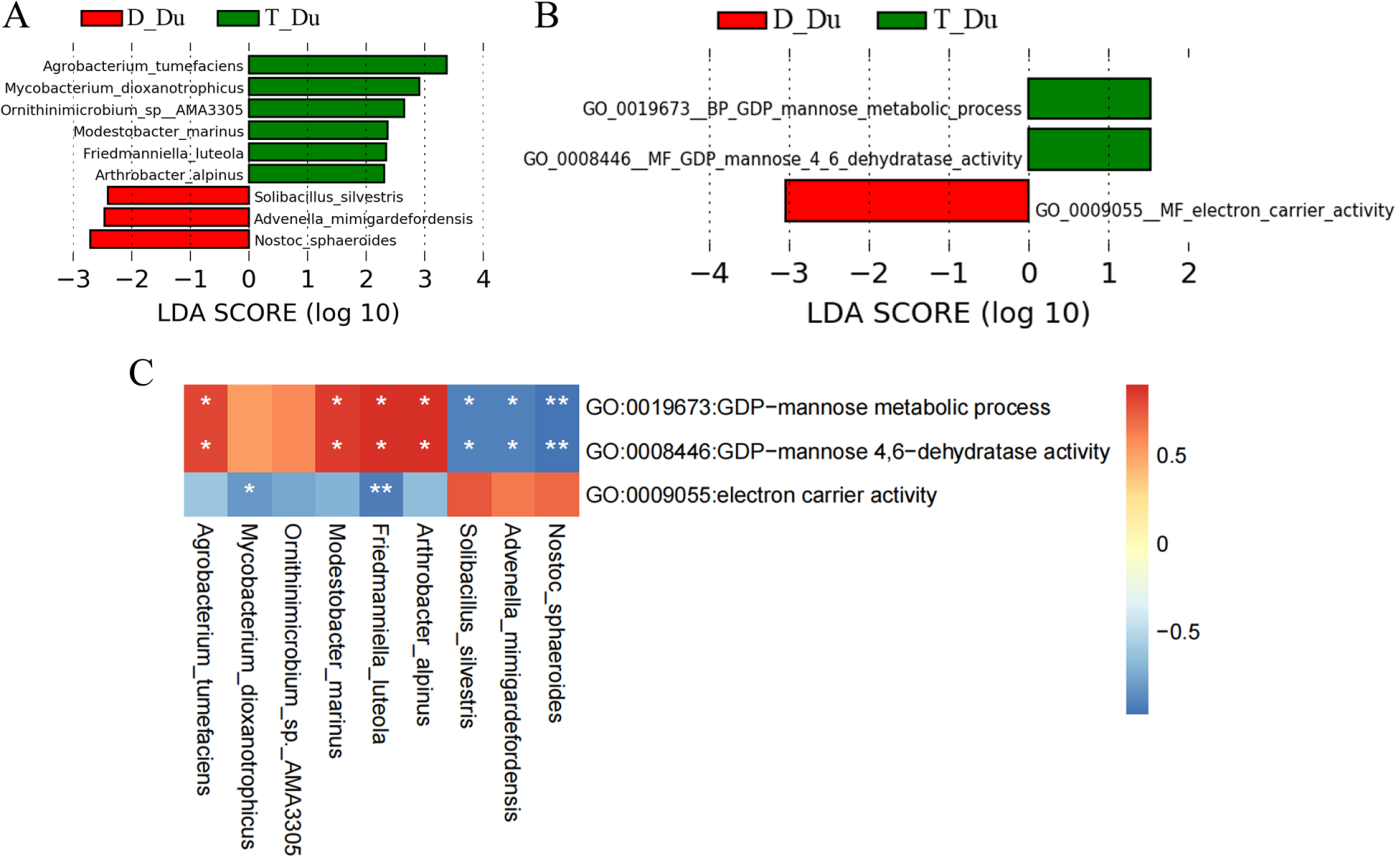
Differences in duodenal microbial composition and function among two sheep breeds from metagenomic data. **A** LEfSe Analysis of duodenal microbiota between the two breeds at the species level. GO differential terms (**B**) of two sheep breeds. **C** Heatmap of correlation between duodenal microbiota and differential pathways. D: Dorper sheep, T: Tan sheep, L: rumen, S: duodenum, J: colon

In the duodenal, 9 bacterial species exhibited significant differences between the two breeds. *Agrobacterium tumefaciens*, *Mycobacterium dioxanotrophicus*, *Ornithinimicrobium sp. AMA3305*, and *Modestobacter marinus* were notably higher in the duodenum of Tan sheep, while *Solibacillus Silvestris*, *Advenella Mimigardefordensis*, and *Nostoc Sphaeroides* were prominently enriched in Dorper sheep (Fig. 4A).

The analysis of Gene Ontology (GO) terms identified 3 distinct pathways (Fig. 4B). GDP-mannose metabolism process and GDP-mannose 4,6-dehydrase activity were more pronounced in Tan sheep. Additionally, mannose-related pathways were significantly positively correlated with *Agrobacterium tumefaciens*, *Modestobacter marinus*, *Friedmanniella luteola, and Arthrobacter alpinus* (Fig. 4C). It was negatively correlated with *Solibacillus silvestris*, *Advenella mimigardefordensis*, *and Nostoc sphaeroides*.

Further investigation into the functional abilities of the gastrointestinal flora involved mapping the microbial gene catalog using metagenomic sequencing data onto the Carbohydrate-Active enZYmes database (CAZy). In the duodenal microbiome of Tan sheep, we discovered a single CAZyme that was substantially more abundant and was predominantly associated with mannose metabolism (Fig. S3). These findings imply that the gastrointestinal bacteria species in the duodenum of Tan sheep may contribute to factors related to mannose metabolism.

### Differences in colonic microbial composition and function between Tan sheep and Dorper sheep

**Fig. 5:**
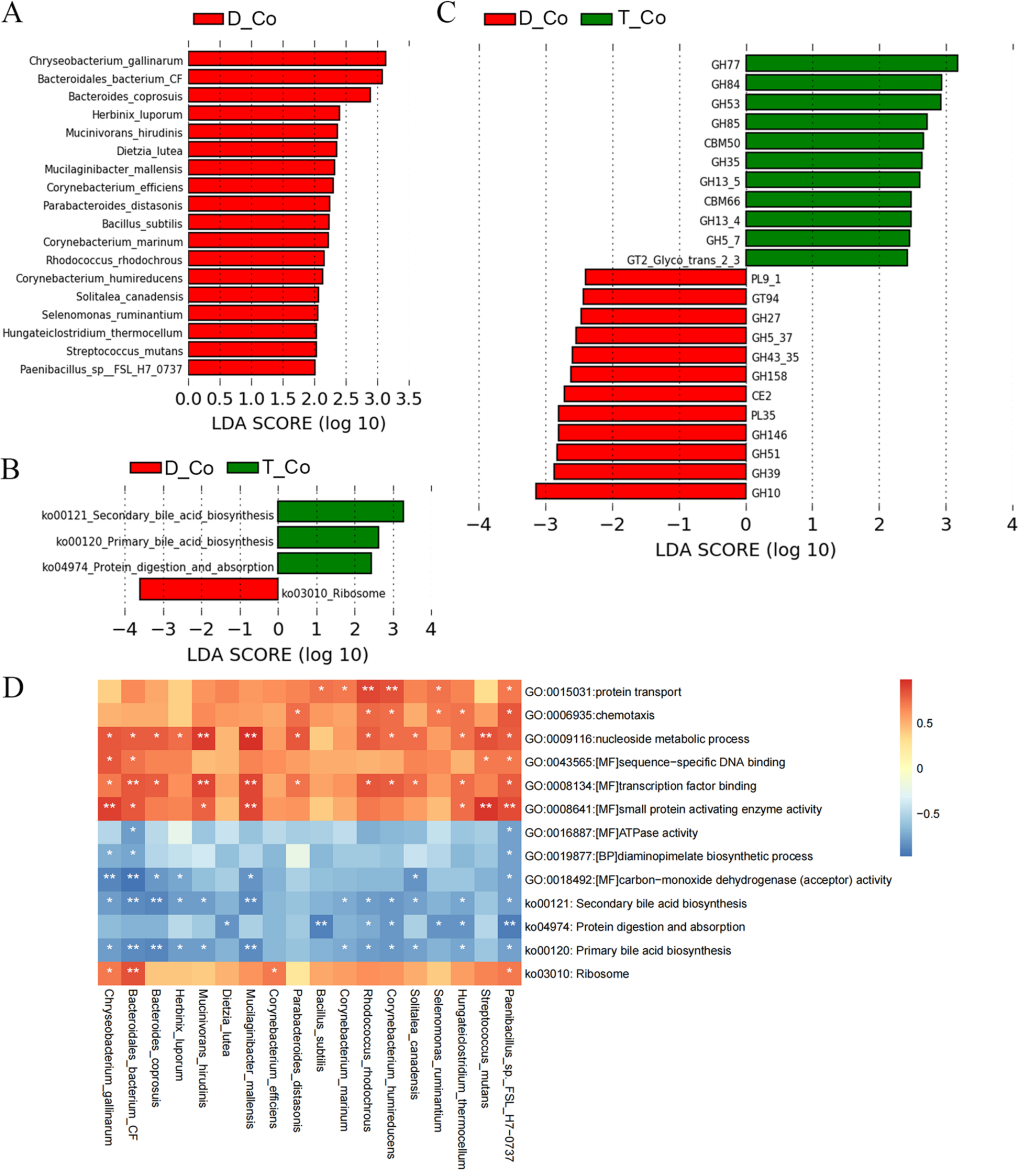
Differences in colonic microbial composition and function between two sheep breeds from metagenomic data. **A** LEfSe Analysis of colonic microbiota between two sheep breeds at the species level. KEGG differential Pathway (**B**) of the two breeds. **C** Function terms of colonic microbes by CAZy between the two breeds. **D** Heatmap of correlation between colonic microbiota and differential pathways. D: Dorper sheep, T: Tan sheep, L: rumen, S: duodenum, J: colon

Eighteen bacterial species were discovered to be considerably different between the two breeds in the colon. Particularly, *Chryseobacterium gallinarum*, *Bacteroidales bacterium CF,* and *Bacteroides coprosuis* were observed to have significantly higher abundance in Tan sheep compared to Dorper sheep (Fig. 5A).

The KEGG functional analysis revealed enriched pathways in Tan sheep such as Secondary bile acid biosynthesis, Nucleotide excision repair, and Primary bile Acid biosynthesis (Fig. 5B). On the other hand, GO terms analysis demonstrated that pathways related to Aspartate carbamoyltransferase activity, Glycine Hydroxymethyltransferase activity, nucleoside metabolic process, and others were significantly enriched in Dorper sheep. Tan sheep exhibited more enrichment pathways in lipid metabolism compared to Dorper sheep (Fig. S4). Correlation analysis explored the relationship between colonic bacteria species and pathways. Notably, *Bacteroidales bacterium CF*, *Bacteroides Coprosuis,* and *Mucilaginibacter mallensis* showed a significantly negative correlation with bile acid synthesis, while displaying a significantly positive correlation with transcription factor binding and nucleoside metabolism (Fig. 5D).

The CAZyme profiles of two sheep breeds in the colonic microbiome were also examined. Between the two sheep kinds, there were substantial differences in the abundances of 23 CAZymes. Among these, 11 CAZymes were abundant in Tan sheep and participated in the metabolism of cellulose, galactose, and amylase. On the other hand, xylan, chondroitin, and arabinose metabolism were predominantly linked to the 12 CAZymes that had greater abundance in the colonic microbiota of Dorper sheep (Fig. 5C). The contribution of various bacteria to the observed alterations in CAZymes was revealed by examining the correlation between the bacterial species and CAZymes (Fig. S5).

### Relationship between fatty acid content of longissimus muscle and microbiota of different gastrointestinal segments

**Fig. 6:**
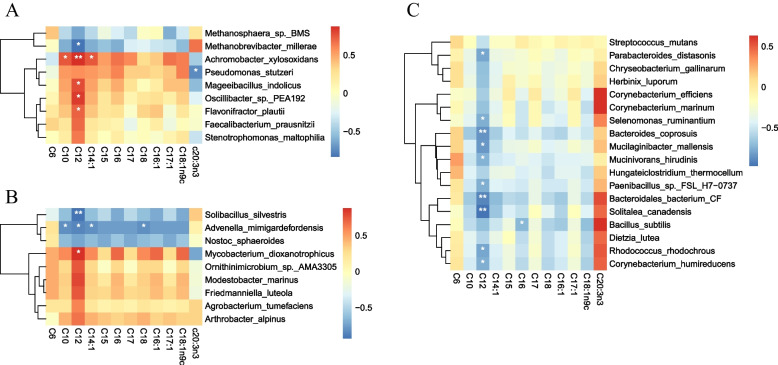
Spearman’s correlations between different microbiomes of the rumen (**A**), duodenum (**B**), and colon (**C**) and fatty acid content of longissimus muscle. The heat maps displayed significant correlations. The correlation values have a direct relationship with the color intensity

To investigate the potential relationship between gastrointestinal microbiota and meat quality traits, we performed a Spearman correlation analysis on the different microbiota present in different gastrointestinal tracts and fatty acid content. The results revealed interesting associations. Specifically, within the rumen, *Achromobacter xylosoxidans*, *Oscillibacter sp. PEA192*, and *Mageeibacillus indolicus* showed significant positive correlations with C12:0 fatty acid, while *Methanobrevibacter millerae* displayed a negative correlation with C12:0 (Fig. 6A). Moving to the duodenum, *Solibacillus silvestris,* and *Advenella mimigardefordensis* exhibited significant negative correlations with C12:0, whereas *Mycobacterium dioxanotrophicus* showed a positive correlation with C12:0 (Fig. 6B). Furthermore, in the colon, several bacteria, including *Bacteroidales bacterium CF*, *Bacteroides coprosuis*, and *Solitalea canadensis* showed significant negative correlations with C12:0 (Fig. 6C). Additionally, *Bacillus subtilis* was found to be negatively correlated with C16:0. These findings provide insight into the potential impact of certain bacteria on the fatty acid and thus meat quality.

## Discussion

The gastrointestinal tract is a multi-organ system with great regional diversity [31]. The fecal microbiome was the major concern of earlier microbiota research. However, the abundance of each segment’s gut microbiota can be qualitatively but not quantitatively represented by feces [32].

The rumen is the main organ for fermenting and digesting cellulose. The large intestine is mostly used for nutrition and water absorption, while the small intestine is primarily used for digesting and absorption. We found the Alpha diversity of the rumen and large intestine was greater than that of the small intestine. One study showed the distal portion of the gut is more diverse and provides more ideal conditions for fermenting refractory polysaccharides, cellulose, and starch [33], which supports our findings. Tan sheep have higher levels of Alpha diversity in each intestine section than Dorper sheep. This indicates that the microbial community of Tan sheep is more diverse and complex.

The rumen primarily contained *Prevotella1*, *Christensenellaceae R-7 group*, *Succiniclasticum*, and *Rikenellaceae RC9 gut group* as the dominant bacteria at the genus level. *Prevotella* can break down plant proteins, starch polysaccharides, and cellulose to create short-chain fatty acids like propionic [34, 35]. *Christensenellaceae R-7 group* enhanced rumen growth and boosted food absorption and digestion [36, 37] The high abundance of these bacteria in the rumen may be linked to the rumen's fiber fermentation and protein and starch breakdown. The foregut primarily contained *Ruminococcus2*, *Lachnospiraceae NK3A20 group, and Christensenellaceae R-7 group.* Among them, *Lachnospiraceae* are engaged in the metabolism of carbohydrates, producing acetic acid and butyric acid, which give the host energy [38]. The significantly abundant bacteria in the hindgut mainly included *Ruminococcaceae*, *Christensenellaceae,* and *Rikenellaceae RC9 gut group*. A study showed that *Ruminococcacea* is related to the degradation of fiber and starch in ruminants [39]. These communities may contribute to further fermentation of feed. The gastrointestinal segments had various enrichment pathways for microbial populations, which may have resulted from distinct gastrointestinal roles.

Previous research by our team has shown that the muscle fiber ratio [41] and fatty acid content [42] of Tan sheep and Duper sheep are significantly different. The gut microbes and host DNA communicate with one another. The rumen, duodenum, and colon microbiotas' impact on the contents of fatty acids allowed us to identify the specific microorganisms that are essential to this relationship. Our results showed that 16 microbial species may play a crucial role in the C12:0 contents of muscle. Of these, 5 were found in the rumen, 3 were in the duodenum, and 8 were in the colon. It indicates that C12:0 may be the bridge between intestinal microbiota and the meat quality of Tan Sheep.

In the study, *Oscillibacter sp. PEA192*, Mageeibacillus indolicus, and *Flavonifractor plautii* belonging to the family Oscillospiraceae were positively correlated with C12:0. Oscillospiraceae is a gram-positive bacterium, which can not only ferment complex plant carbohydrates to produce butanoic acid but also makes use of gluconate [40]. Previous studies have found that butyric-producing bacteria have a long-term intervention in obesity, and have shown beneficial effects on host glucose, lipid metabolism, and gut microbial composition [41]. In addition, our results demonstrated that the differential bacteria were primarily concentrated in the metabolism of xylan, glucan, mannose, and amylase. So, we speculate that Oscillospiraceae may change the content of lauric acid in muscle.

*Methanobrevibacter millerae* belong to the genus *Methanobrevibacter.* As shown in a study, thin Landrace pigs had more copies of the methanogen mcrA gene and a wider variety of methanogens than fat Erhualian pigs [42]. This study found a statistically negative connection between the abundance of *Methanobrevibacter millerae* and C12:0 in Dorper sheep compared to Tan sheep. It indicates that methane-producing bacteria are related to fat deposition and fatty acid content in muscle. A common link between *Achromobacter xylosoxidans* and cystic fibrosis of the lung shows that it is an emerging pathogen [43]. According to the current study, *Achromobacter xylosoxidans* may benefit sheep gut because of its large abundance and significantly positive connection with C12:0.

*Bacteroidales bacterium CF*, *Bacteroides coprosuis*, *Mucinivorans hirudinis*, and *Parabacteroides distasonis* belonging to order Bacteroidales were negatively correlated with C12:0. Bacteroides are polysaccharide-degrading consortia members that aid in the release of energy from dietary starch and fiber as well as the production of SCFAs like propionate [44]. Propionate can activate the PPAR-γ signaling pathway in the liver and adipose tissue and affect adipose metabolism [45]. Our results showed that the distinct bacteria were primarily concentrated in the metabolism of cellulose, galactose, and bile acid. At the same time, bile acid is both a detergent that promotes digestion and absorption of dietary fats and a hormone that activates different receptors. Fat metabolism requires bile acid and its interaction with gut bacteria [46]. Accordingly, we postulate that Bacteroidales in sheep guts could change the C12:0 content of longissimus muscle through their metabolic pathway.

## Conclusion

In this study, we explored the differences in the distribution and functional patterns of gastrointestinal microbiota between Tan sheep and Dorper sheep using 16Sr DNA and metagenomic techniques. 36 microbial species were found different, such as *Achromobacter xylosoxidans*, *and Oscillibacter sp. PEA192*, *Solibacillus silvestris*, *Bacteroidales bacterium CF*, and *Bacteroides coprosuis.* Among these, 16 bacteria may influence C12:0 of the longissimus muscle through their metabolic pathways. Overall, our findings imply that target attributes may be altered by modifying the microbes in the gut and offer a more thorough understanding of the role of the microbiota in various gut segments on meat quality traits. Due to the limitations of the sample population, the results need to be further verified by experiments in large groups.

### Supplementary Information

**Additional file 1: Table S1.** The sequence information for each sample. **Table S2.** The Alpha diversity in Tan sheep and Dorper sheep. **Table S3.** Difference analysis of dominant bacteria genera in Tan and Dorper sheep's different gastrointestinal segments.

**Additional file 2: Fig. S1.** The log-transformed LDA scores illustrate significant KEGG functions in rumen of Tan Sheep and Dorper Sheep. **Fig. S2.** Correlation analysis between the species and CAZymes in rumen. **Fig. S3.** Function terms of duodenal microbes by CAZy between Tan Sheep and Dorper Sheep. **Fig. S4.** GO differential terms in microbial function of colon between Tan Sheep and Dorper Sheep. **Fig. S5.** Correlation analysis between the species and CAZymes in colon. **Fig. S6.** LEfSe Analysis of ruminal microbiota among two sheep breeds at the genus level. **Fig. S7.** LEfSe Analysis of duodenal microbiota between the two breeds at the genus level. **Fig. S8.** LEfSe Analysis of jejunal microbiota between the two breeds at the genus level. **Fig. S9.** LEfSe Analysis of colonic microbiota between the two breeds at the genus level. **Fig. S10.** LEfSe Analysis of cecal microbiota between the two breeds at the genus level.

## Data Availability

The Metagenomic data available were deposited in the NCBI Sequence Read Archive (SRA) under the accession number: PRJNA937780.
