# Supplementary material for: Specific gastrointestinal microbiota profiles in Chinese Tan sheep are associated with lauric acid content in muscle
Source: BMC Microbiol. 2023 Nov 8;23:331. doi: 10.1186/s12866-023-03079-2 (PMC10631117; doi:10.1186/s12866-023-03079-2)
Supplement: Supplementary file 2 — Additional file 2: Fig. S1. The log-transformed LDA scores illustrate significant KEGG functions in rumen of Tan Sheep and Dorper Sheep. Fig. S2. Correlation analysis between the species and CAZymes in rumen. Fig. S3. Function terms of duodenal microbes by CAZy between Tan Sheep and Dorper Sheep. Fig. S4. GO differential terms in microbial function of colon between Tan Sheep and Dorper Sheep. Fig. S5. Correlation analysis between the species and CAZymes in colon. Fig. S6. LEfSe Analysis of ruminal microbiota among two sheep breeds at the genus level. Fig. S7. LEfSe Analysis of duodenal microbiota between the two breeds at the genus level. Fig. S8. LEfSe Analysis of jejunal microbiota between the two breeds at the genus level. Fig. S9. LEfSe Analysis of colonic microbiota between the two breeds at the genus level. Fig. S10. LEfSe Analysis of cecal microbiota between the two breeds at the genus level. [file 12866_2023_3079_MOESM2_ESM.docx]

**SUPPLEMENTARY INFORMATION**

**FIGURES**

**
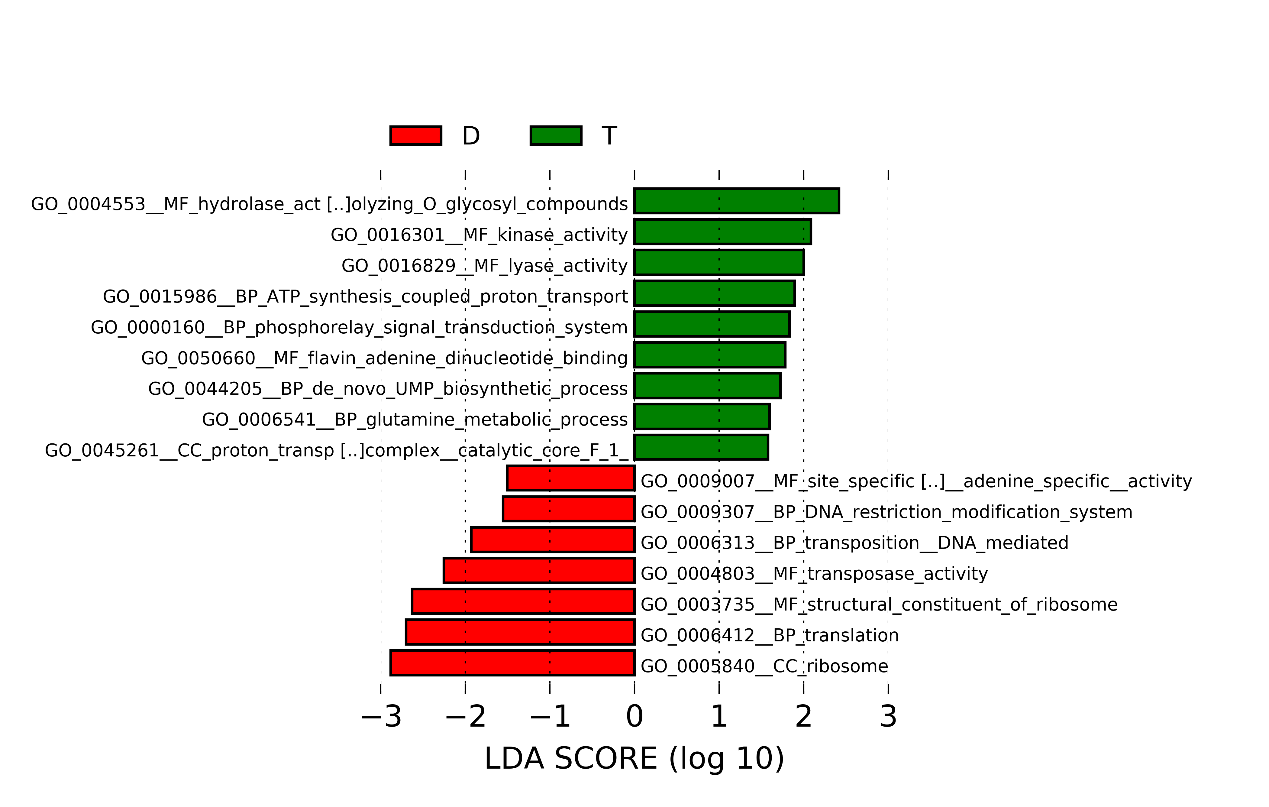
**

Fig. S1 The log-transformed LDA scores illustrate significant KEGG functions in rumen of Tan Sheep and Dorper Sheep.


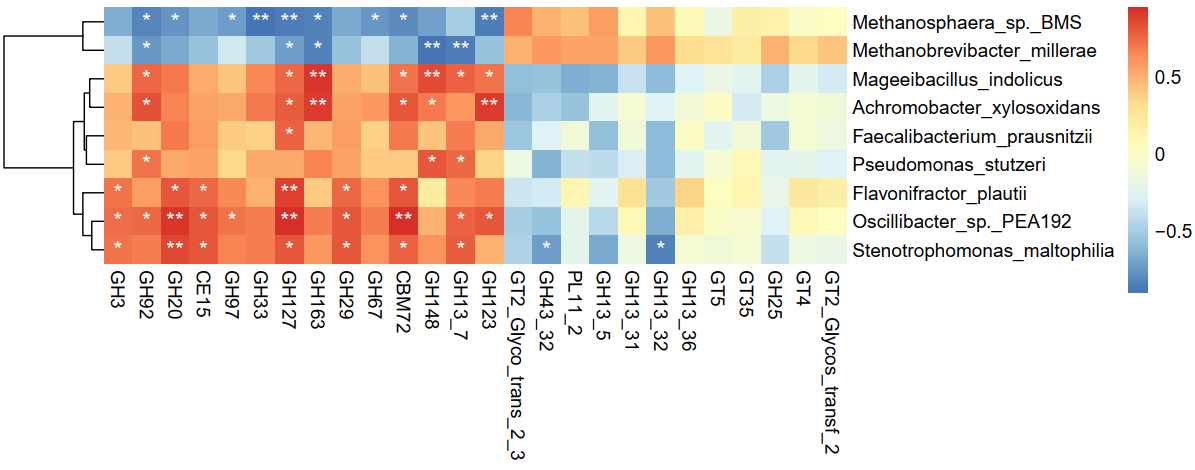


Fig. S2 Correlation analysis between the species and CAZymes in rumen.


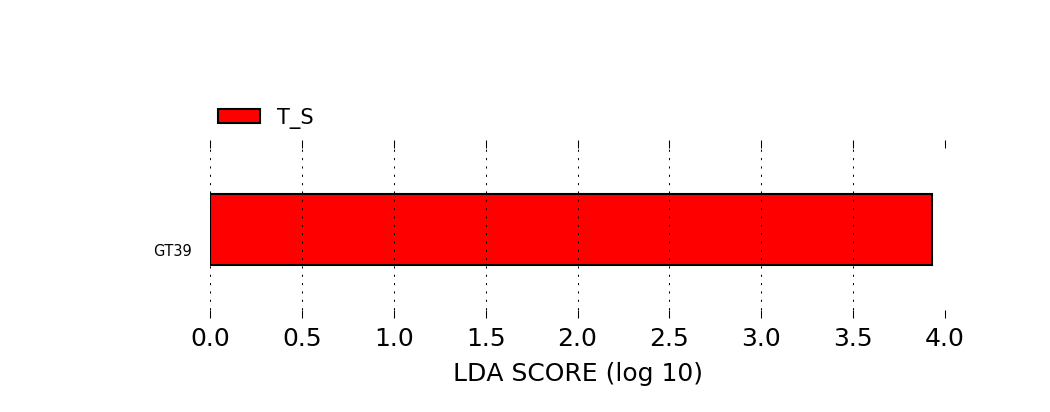


Fig. S3 Function terms of duodenal microbes by CAZy between Tan Sheep and Dorper Sheep.


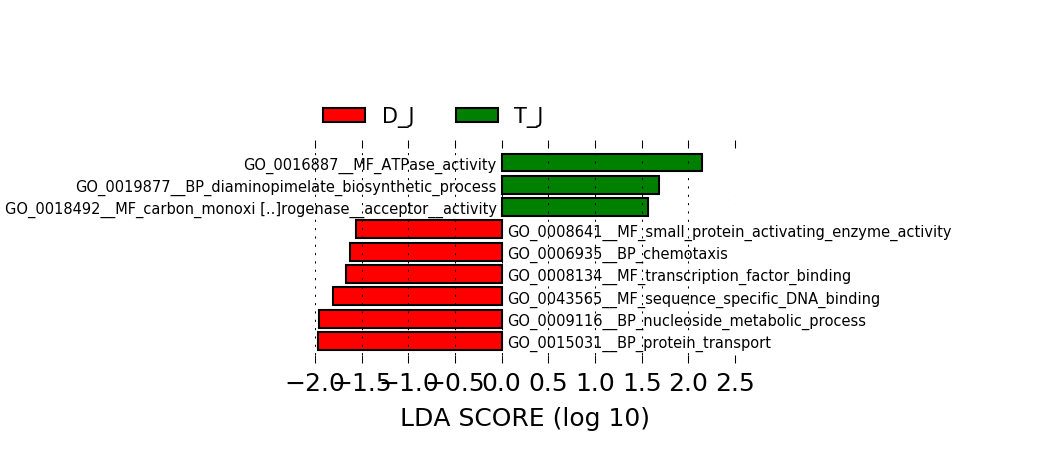


Fig. S4 GO differential terms in microbial function of colon between Tan Sheep and Dorper Sheep.


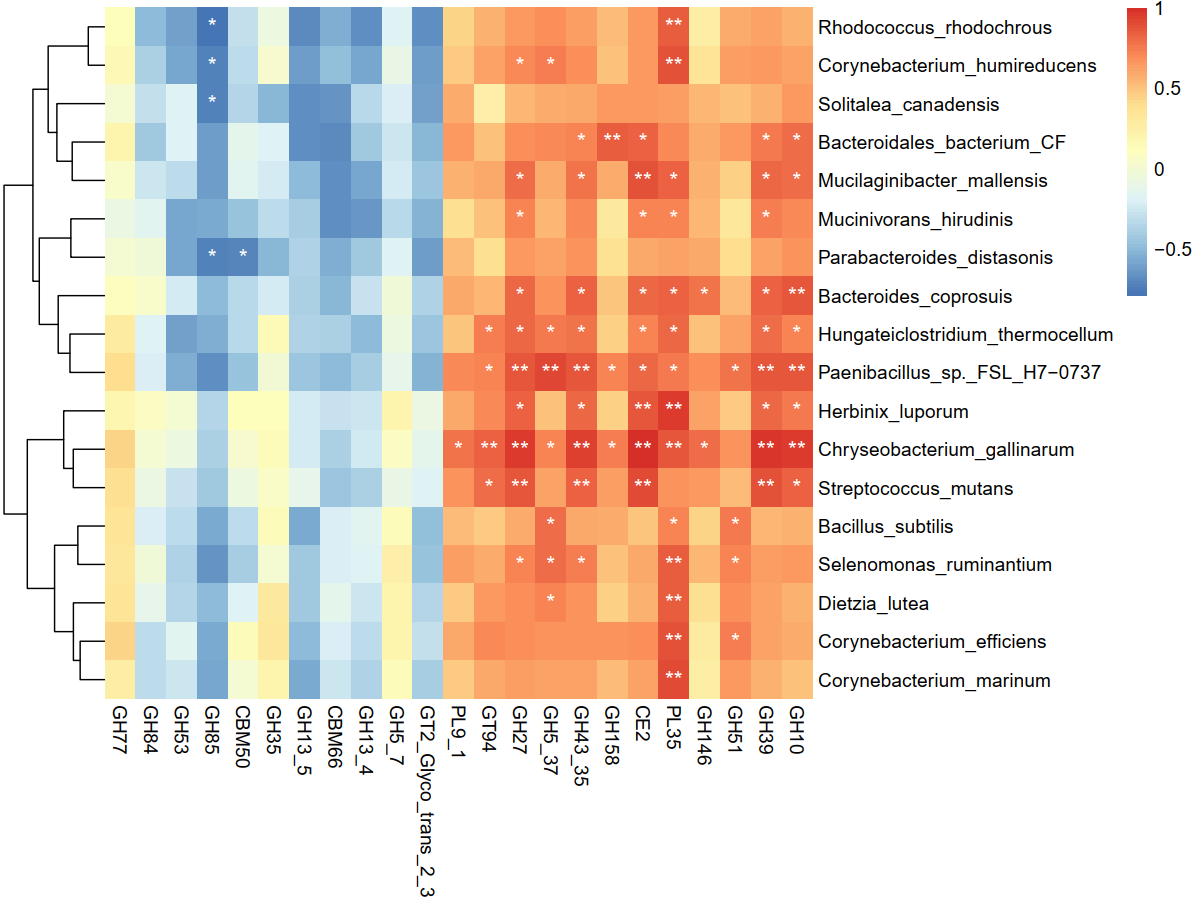


Fig. S5 Correlation analysis between the species and CAZymes in colon.


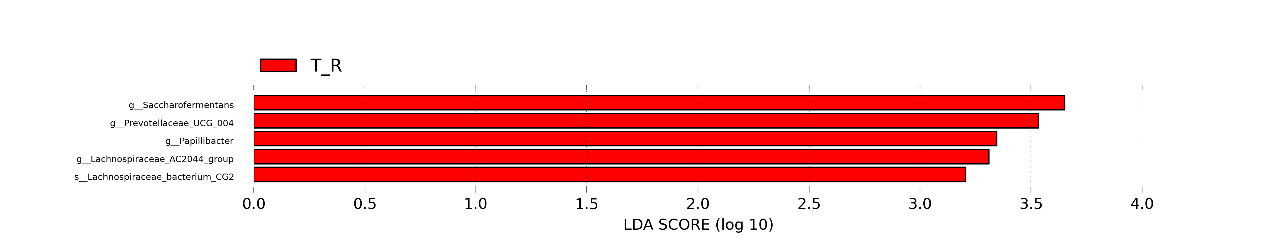


Fig.S6 LEfSe Analysis of ruminal microbiota among two sheep breeds at the genus level.


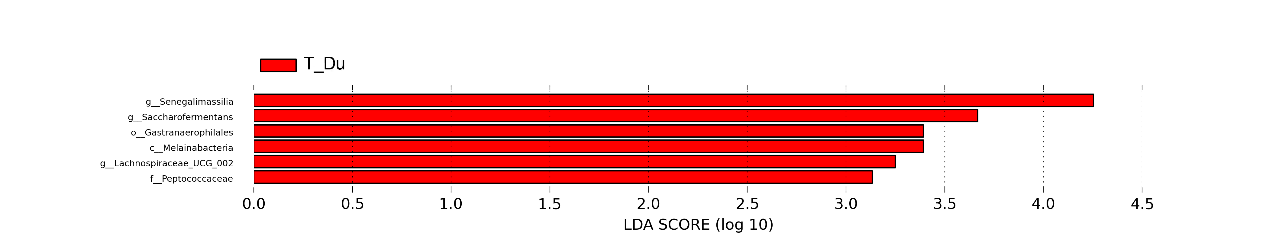


Fig.S7 LEfSe Analysis of duodenal microbiota between the two breeds at the genus level.


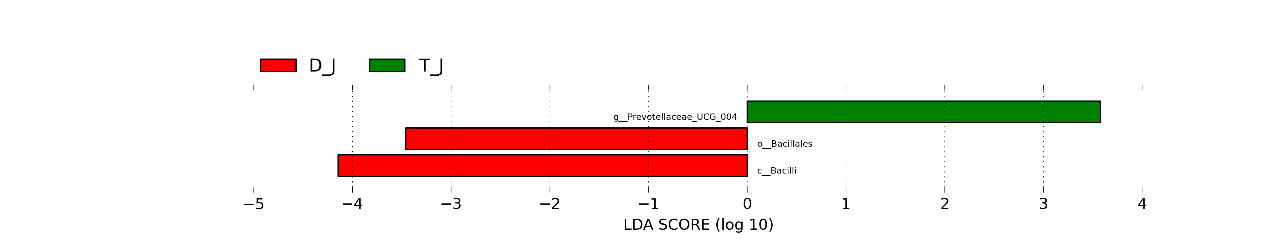


Fig.S8 LEfSe Analysis of jejunal microbiota between the two breeds at the genus level.


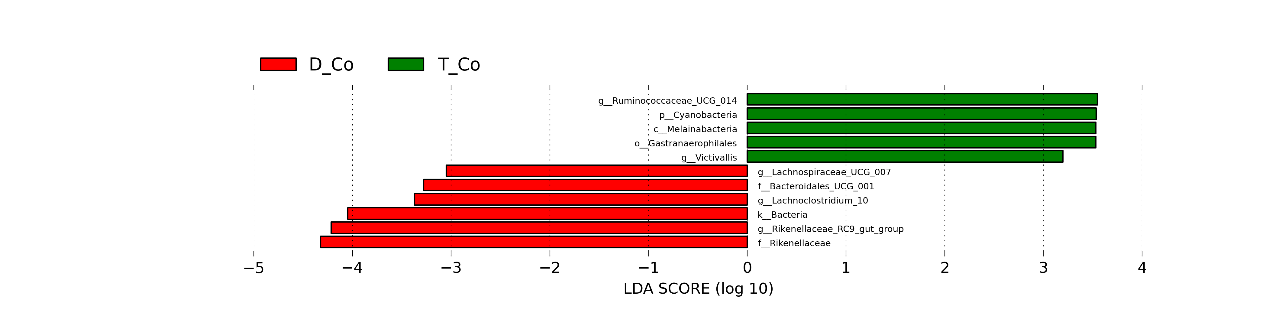


Fig.S9 LEfSe Analysis of colonic microbiota between the two breeds at the genus level.


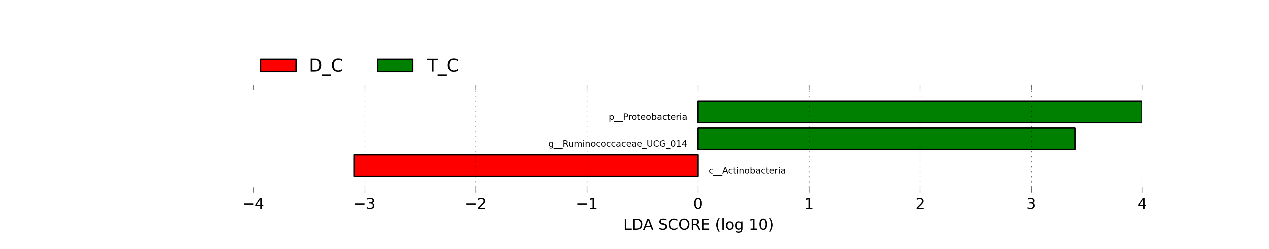


Fig.S10 LEfSe Analysis of cecal microbiota between the two breeds at the genus level.
